# Quantum Correlation in Squeezed Generalized Amplitude Damping Channels with Memory

**DOI:** 10.1038/s41598-019-40652-0

**Published:** 2019-03-11

**Authors:** Youngmin Jeong, Hyundong Shin

**Affiliations:** Department of Electronic Engineering, Kyung Hee University, Yongin-si, 17104 Korea

## Abstract

A squeezed generalized amplitude damping (SGAD) channel is a quantum channel that models a general noise process incorporating the effects of bath squeezing, dissipation, and decoherence. In this paper, we analyze the dynamics of quantum entanglement and discord in the SGAD channel with memory. By obtaining a stochastic map defining this noisy quantum channel, we derive the concurrence and discord of Werner-like mixed states sent by successive uses of the channel. It is shown that these quantum correlations can be preserved or even generated depending on the initial channel input states, channel parameters, and the degree of channel memory. In particular, the squeezing effect does not contribute to the dynamics of quantum correlation for singlet-like states under correlated noise.

## Introduction

Quantum correlation (e.g., entanglement^1,2^ and discord^3–5^) is a fundamental feature of quantum mechanics, which is known to be at the heart of various potential applications, such as superdense coding, quantum teleportation, and quantum cryptography^6–10^. However, the quantum correlation is very fragile and broken by unexpected and unwanted interactions with an environment–referred as quantum *noise*. Therefore, the evolution of quantum correlation in various noisy environments has been a topic of importance in a field of quantum information processing and quantum computation^11–13^.

An amplitude damping channel is a quantum channel that models a physical process such as spontaneous emission or energy dissipation at zero temperature^14^. More generally, a quantum noise process due to dissipative interactions with a purely thermal bath is modeled by a generalized amplitude damping channel, which is one of the most important quantum channels and describes the dissipation effect at finite temperature^15^. This noisy quantum channel is further extended to a squeezed generalized amplitude damping (SGAD) channel by taking into account a squeezed thermal bath^16^. The SGAD channel incorporates the both effects of dissipation at finite temperature and bath squeezing^17–20^. The squeezed thermal bath can suppress quantum decoherence^18^, while it is unable to help in preserving quantum entanglement^19,20^.

A noisy quantum channel is defined by a stochastic map^17^

1 $${{\rm{\Phi }}}_{1}:\rho \mapsto {{\rm{\Phi }}}_{1}(\rho ),$$

which is completely positive trace-preserving and transforms a quantum state described by a density operator ***ρ*** into a quantum state Φ_1_(*ρ*). The density operator ***ρ*** satisfies a master equation^21^

2 $$\frac{d\rho }{dt}=\frac{1}{i\hslash }[H,\rho ]+ {\mathcal L} \rho ,$$

where $i=\sqrt{-1}$, *H* is the system Hamiltonian, ${\mathcal L}$ is the Lindblad superoperator, and [⋅,⋅] denotes the commutator. The first term in (2) describes coherent dynamics while the second term accounts for damping mechanisms. The SGAD channel is a general Lindbladian noisy channel in which a quantum system interacts with a bath being initially in a squeezed thermal state under the Markov and Born approximations. The corresponding Lindblad superoperator has the form of^17^

3 $$\begin{array}{c} {\mathcal L} \rho =-\,\frac{{\rm{\Omega }}(n+1)}{2}({\sigma }_{+}{\sigma }_{-}\rho +\rho {\sigma }_{+}{\sigma }_{-}-2{\sigma }_{-}\rho {\sigma }_{+})-\frac{{\rm{\Omega }}n}{2}({\sigma }_{-}{\sigma }_{+}\rho +\rho {\sigma }_{-}{\sigma }_{+}-2{\sigma }_{+}\rho {\sigma }_{-})\\ \,\,\,-{\rm{\Omega }}m({\sigma }_{+}\rho {\sigma }_{+}+{\sigma }_{-}\rho {\sigma }_{-}),\end{array}$$

where ${\sigma }_{+}=\frac{1}{2}({\sigma }_{1}+i{\sigma }_{2})$ and ${\sigma }_{-}=\frac{1}{2}({\sigma }_{1}-i{\sigma }_{2})$ are creation and annihilation operators, respectively; *σ*_*i*_ for *i* = 1, 2, 3 are Pauli *x*, *y*, and *z* operators; *n* is related to the number of thermal photons; *m* < *n* + 1/2 is the squeezing parameter; and Ω is the zero-temperature dissipation rate, associated to the spontaneous emission^14,17,22^. Note that the SGAD channel reduces to the generalized amplitude damping channel (thermal field channel) when *m* = 0 and further to the amplitude damping channel (spontaneous emission) by setting *n* = *m* = 0. As *n* → ∞, Φ_1_(*ρ*) tends to a maximally mixed state.

The simplest model for quantum channel uses is memoryless, that is, the quantum operation describing *N* channel uses is equal to ${\rm{\Phi }}={{\rm{\Phi }}}_{1}^{\otimes N}$. However, some noise process can introduce memory effects among consecutive channel uses^23–25^, leading to ${\rm{\Phi }}\ne {{\rm{\Phi }}}_{1}^{\otimes N}$. The effect of channel memory (or time-correlated noise) was widely explored in various quantum channels^26–36^. It was shown that the channel memory can enhance the classical capacity of a quantum channel by using entangled quantum states rather than separable quantum states^26–33^. The channel memory can also freeze the evolution of quantum correlation between two qubits sent by successive channel uses^34–36^ with avoiding the entanglement sudden death (ESD) phenomenon^37^.

The aim of this paper is to analyze the dynamics of quantum correlation of Werner-like mixed states sent by two consecutive uses of the SGAD channel *with memory*. We first find stochastic maps defining the SGAD channel with uncorrelated and correlated noises. We then provide analytical expressions for the concurrence and discord. The roles of initial channel input states, the degree of channel memory, the number of thermal photons, and bath squeezing on the dynamics of concurrence and discord are investigated by numerical examples.

## Results

### The SGAD Channel with Memory

When the environmental correlation time is longer than the time between two consecutive channel uses, the overall stochastic map Φ for the two channel uses does not obey ${\rm{\Phi }}={{\rm{\Phi }}}_{1}^{\otimes 2}$ due to the channel memory. This effect was verified experimentally in a fiber optic link exhibiting fluctuating birefringence^23^ and in the solid-state implementation of quantum hardware suffering from low frequency noise^24,25^. The stochastic map Φ for two qubits of initial states ${\boldsymbol{\rho }}\in {{\mathbb{C}}}^{4\times 4}$ sent by two consecutive uses of a quantum channel with memory can be written as^29^

4 $${\rm{\Phi }}({\boldsymbol{\rho }})=(1-\mu ){{\rm{\Phi }}}_{{\rm{u}}}({\boldsymbol{\rho }})+\mu {{\rm{\Phi }}}_{{\rm{c}}}({\boldsymbol{\rho }}),$$

where Φ_u_ and Φ_c_ denote the stochastic maps corresponding to uncorrelated (memoryless) and correlated (memory) noise effects of the channel, respectively; and ***μ*** is a degree of channel memory, implying that the noise is correlated with probability ***μ***. The stochastic map Φ_u_(***ρ***) for the SGAD channel with uncorrelated noise is given by (see Methods)

5 $${{\rm{\Phi }}}_{{\rm{u}}}({\boldsymbol{\rho }})={\sum }_{i,j}{{\boldsymbol{A}}}_{i,j}{\boldsymbol{\rho }}{{\boldsymbol{A}}}_{i,j}^{\dagger },$$

where ***A***_*i*,*j*_ = *E*_*i*_ ⊗ *E*_*j*_ and the Kraus operators *E*_*i*_ are

6 $${E}_{1}=(\begin{array}{cc}\sqrt{\frac{n}{2n+1}+{e}^{-2{\rm{\Omega }}(n+\mathrm{1/2})t}\frac{n+1}{2n+1}-{e}^{-{\rm{\Omega }}(n+\mathrm{1/2})t}\,\cosh ({\rm{\Omega }}mt)} & 0\\ 0 & 0\end{array}),$$

7 $${E}_{2}=(\begin{array}{cc}0 & 0\\ \sqrt{\frac{n+1}{2n+1}(1-{e}^{-2{\rm{\Omega }}(n+\mathrm{1/2})t})+{e}^{-{\rm{\Omega }}(n+\mathrm{1/2})t}\,\sinh ({\rm{\Omega }}mt)} & 0\end{array}),$$

8 $${E}_{3}=(\begin{array}{cc}0 & 0\\ 0 & \sqrt{\frac{n+1}{2n+1}+{e}^{-2{\rm{\Omega }}(n+\mathrm{1/2})t}\frac{n}{2n+1}-{e}^{-{\rm{\Omega }}(n+\mathrm{1/2})t}\,\cosh ({\rm{\Omega }}mt)}\end{array}),$$

9 $${E}_{4}=(\begin{array}{cc}\sqrt{{e}^{-{\rm{\Omega }}(n+\mathrm{1/2})t}\,\cosh ({\rm{\Omega }}mt)} & 0\\ 0 & \sqrt{{e}^{-{\rm{\Omega }}(n+\mathrm{1/2})t}\,\cosh ({\rm{\Omega }}mt)}\end{array}),$$

10 $${E}_{5}=(\begin{array}{cc}0 & \sqrt{{e}^{-{\rm{\Omega }}(n+\mathrm{1/2})t}\,\sinh ({\rm{\Omega }}mt)}\\ \sqrt{{e}^{-{\rm{\Omega }}(n+\mathrm{1/2})t}\,\sinh ({\rm{\Omega }}mt)} & 0\end{array}),$$

11 $${E}_{6}=(\begin{array}{cc}0 & \sqrt{\frac{n}{2n+1}(1-{e}^{-2{\rm{\Omega }}(n+\mathrm{1/2})t})+{e}^{-{\rm{\Omega }}(n+\mathrm{1/2})t}\,\sinh ({\rm{\Omega }}mt)}\\ 0 & 0\end{array})\mathrm{.}$$

The stochastic map Φ_c_(***ρ***) for the SGAD channel with correlated noise has the Kraus decomposition as follows (see Methods):

12 $${{\rm{\Phi }}}_{{\rm{c}}}({\boldsymbol{\rho }})={\sum }_{k}{{\boldsymbol{B}}}_{k}{\boldsymbol{\rho }}{{\boldsymbol{B}}}_{k}^{\dagger },$$

where the Kraus operators ***B***_*k*_ are given by

13 $${{\boldsymbol{B}}}_{1}=(\begin{array}{cccc}\sqrt{{e}^{-{\rm{\Omega }}(n+1)t}} & 0 & 0 & 0\\ 0 & 1 & 0 & 0\\ 0 & 0 & 1 & 0\\ 0 & 0 & 0 & \sqrt{{e}^{-{\rm{\Omega }}nt}}\end{array}),$$

14 $${{\boldsymbol{B}}}_{2}=(\begin{array}{cccc}0 & 0 & 0 & 0\\ 0 & 0 & 0 & 0\\ 0 & 0 & 0 & 0\\ \sqrt{\frac{n+1}{2n+1}(1-{e}^{-2{\rm{\Omega }}(n+\mathrm{1/2})t})+{e}^{-{\rm{\Omega }}(n+\mathrm{1/2})t}\,\sinh ({\rm{\Omega }}mt)} & 0 & 0 & 0\end{array}),$$

15 $${{\boldsymbol{B}}}_{3}=(\begin{array}{cccc}0 & 0 & 0 & \sqrt{\frac{n}{2n+1}(1-{e}^{-2{\rm{\Omega }}(n+\mathrm{1/2})t})+{e}^{-{\rm{\Omega }}(n+\mathrm{1/2})t}\,\sinh ({\rm{\Omega }}mt)}\\ 0 & 0 & 0 & 0\\ 0 & 0 & 0 & 0\\ 0 & 0 & 0 & 0\end{array}),$$

16 $${{\boldsymbol{B}}}_{4}=(\begin{array}{cccc}\sqrt{\frac{n}{2n+1}+\frac{n+1}{2n+1}{e}^{-2{\rm{\Omega }}(n+\mathrm{1/2})t}-{e}^{-{\rm{\Omega }}(n+\mathrm{1/2})t}(\cosh ({\rm{\Omega }}mt)-1)-{e}^{-{\rm{\Omega }}(n+1)t}} & 0 & 0 & 0\\ 0 & 0 & 0 & 0\\ 0 & 0 & 0 & 0\\ 0 & 0 & 0 & 0\end{array}),$$

17 $${{\boldsymbol{B}}}_{5}=(\begin{array}{cccc}0 & 0 & 0 & 0\\ 0 & 0 & 0 & 0\\ 0 & 0 & 0 & 0\\ 0 & 0 & 0 & \sqrt{\frac{n+1}{2n+1}+\frac{n}{2n+1}{e}^{-2{\rm{\Omega }}(n+\mathrm{1/2})t}-{e}^{-{\rm{\Omega }}(n+\mathrm{1/2})t}(\cosh ({\rm{\Omega }}mt)-1)-{e}^{-{\rm{\Omega }}nt}}\end{array}),$$

18 $${{\boldsymbol{B}}}_{6}=(\begin{array}{cccc}\sqrt{{e}^{-{\rm{\Omega }}(n+\mathrm{1/2})t}(\cosh ({\rm{\Omega }}mt)-1)} & 0 & 0 & 0\\ 0 & 0 & 0 & 0\\ 0 & 0 & 0 & 0\\ 0 & 0 & 0 & \sqrt{{e}^{-{\rm{\Omega }}(n+\mathrm{1/2})t}(\cosh ({\rm{\Omega }}mt)-1)}\end{array}),$$

19 $${{\boldsymbol{B}}}_{7}=(\begin{array}{cccc}0 & 0 & 0 & i\sqrt{{e}^{-{\rm{\Omega }}(n+\mathrm{1/2})t}\,\sinh ({\rm{\Omega }}mt)}\\ 0 & 0 & 0 & 0\\ 0 & 0 & 0 & 0\\ i\sqrt{{e}^{-{\rm{\Omega }}(n+\mathrm{1/2})t}\,\sinh ({\rm{\Omega }}mt)} & 0 & 0 & 0\end{array})\mathrm{.}$$

In contrast to the Kraus operators ***A***_*i*,*j*_ for the uncorrelated action Φ_u_, the Kraus operators ***B***_*k*_ for the correlated action Φ_c_ are not in general tensorial forms (see Supplementary Material).

### Dynamics of Quantum Correlation

Quantum correlation can be classified into two categories: entanglement and information-theoretic measures–*discord*. In contrast to entanglement^3,5^, the quantum discord can be positive even for certain separable mixed states^9,10^. We now study the dynamics of the concurrence and discord of Werner-like mixed states sent by two successive uses of the SGAD channel with memory.

#### Werner-Like Mixed States

We consider Werner-like mixed states ${{\boldsymbol{\rho }}}_{\pm }\in {{\mathbb{C}}}^{4\times 4}$ as initial input states for two qubits:

20 $${{\boldsymbol{\rho }}}_{\pm }=\varepsilon |{{\boldsymbol{\psi }}}_{\pm }\rangle \langle {{\boldsymbol{\psi }}}_{\pm }|+\frac{1-\varepsilon }{4}{I}_{4},$$

where ***ε*** ∈ [0, 1] denotes the purity of the initial state, *I*_4_ is the 4 × 4 identity matrix, and

21 $$|{{\boldsymbol{\psi }}}_{+}\rangle =\sqrt{1/2}(|00\rangle +|11\rangle ),$$

22 $$|{{\boldsymbol{\psi }}}_{-}\rangle =\sqrt{1/2}(|01\rangle -|10\rangle ),$$

correspond to the Bell-like entangled two-qubit states^38^. The Werner-like states ***ρ***_±_ are entangled when *ε* > 1/3, otherwise ***ρ***_±_ are separable states. Specifically, we call ***ρ***_−_ as a singlet-like state.

#### Quantum Entanglement

For any density matrix ${\boldsymbol{\rho }}\in {{\mathbb{C}}}^{4\times 4}$ for two-qubit states, the concurrence, denoted by ${\mathfrak{C}}({\boldsymbol{\rho }})$, can be computed as^39^

23 $${\mathfrak{C}}({\boldsymbol{\rho }})={[\sqrt{{\nu }_{1}}-\sqrt{{\nu }_{2}}-\sqrt{{\nu }_{3}}-\sqrt{{\nu }_{4}}]}^{+},$$

where *ν*_1_ > *ν*_2_ > *ν*_3_ > *ν*_4_ are the eigenvalues of the density matrix ${\boldsymbol{\rho }}{\sigma }_{2}^{\otimes 2}{{\boldsymbol{\rho }}}^{\ast }{\sigma }_{2}^{\otimes 2}$ in descending order and [*x*]^+^ = max{*x*, 0} denotes a positive part of *x*.

Let ***ρ***_±_(*t*) = Φ(***ρ***_±_) be the output state of the channel. Then, these output Werner-like states ***ρ***_±_(*t*) belong to the family of the so-called X-states^40^ (see Supplementary Material) as follows:

24 $${{\boldsymbol{\rho }}}_{\pm }(t)=(\begin{array}{cccc}{\rho }_{11}^{\pm }(t) & 0 & 0 & \frac{1}{2}{\rho }_{14}(t)\pm \frac{1}{2}{\rho }_{14}(t)\\ 0 & {\rho }_{22}^{\pm }(t) & \frac{1}{2}{\rho }_{23}(t)\mp \frac{1}{2}{\rho }_{23}(t) & 0\\ 0 & \frac{1}{2}{\rho }_{23}^{\ast }(t)\mp \frac{1}{2}{\rho }_{23}^{\ast }(t) & {\rho }_{33}^{\pm }(t) & 0\\ \frac{1}{2}{\rho }_{14}^{\ast }(t)\pm \frac{1}{2}{\rho }_{14}^{\ast }(t) & 0 & 0 & {\rho }_{44}^{\pm }(t)\end{array}),$$

in the computational basis $|00\rangle$, $|01\rangle$, $|10\rangle$ and $|11\rangle$ for two qubits where tr(***ρ***_±_(*t*)) = 1. The nonzero elements of ***ρ***_±_(*t*) are given by

25 $${\rho }_{11}^{\pm }(t)=\frac{(2n+{\rm{\Lambda }}(t))(1\pm \varepsilon )}{4(2n+1)}\mu +\frac{4{n}^{2}+4n{\rm{\Lambda }}(t)+(1\pm \varepsilon {(1+2n)}^{2}){{\rm{\Lambda }}}^{2}(t)}{4{(2n+1)}^{2}}(1-\mu ),$$

26 $${\rho }_{22}^{\pm }(t)=\frac{1\mp \varepsilon }{4}\mu +\frac{1}{4}(1\mp \varepsilon {{\rm{\Lambda }}}^{2}(t)-{(\frac{1-{\rm{\Lambda }}(t)}{2n+1})}^{2})(1-\mu ),$$

27 $${\rho }_{33}^{\pm }(t)=\frac{1\mp \varepsilon }{4}\mu +\frac{1}{4}(1\mp \varepsilon {{\rm{\Lambda }}}^{2}(t)-{(\frac{1-{\rm{\Lambda }}(t)}{2n+1})}^{2})(1-\mu ),$$

28 $${\rho }_{44}^{\pm }(t)=\frac{(2n+2-{\rm{\Lambda }}(t))(1\pm \varepsilon )}{4(2n+1)}\mu +\frac{4{(n+1)}^{2}-4(n+1){\rm{\Lambda }}(t)+(1\pm \varepsilon {(2n+1)}^{2}){{\rm{\Lambda }}}^{2}(t)}{4{(2n+1)}^{2}}(1-\mu ),$$

29 $${\rho }_{14}(t)=\frac{\varepsilon \sqrt{{\rm{\Lambda }}(t)}{e}^{-{\rm{\Omega }}mt}}{2}\mu +\frac{\varepsilon \,\cosh (2{\rm{\Omega }}mt){\rm{\Lambda }}(t)}{2}(1-\mu ),$$

30 $${\rho }_{23}(t)=\frac{\varepsilon }{2}\mu +\frac{\varepsilon \,\cosh (2{\rm{\Omega }}mt){\rm{\Lambda }}(t)}{2}(1-\mu ),$$

where Λ(*t*) = *e*^−Ω(2*n*+1)*t*^ is the damping parameter. In what follows, we drop the function of time in these nonzero elements for notational simplicity. Using the concurrence expression for the X-state^39^, we obtain

31 $${\mathfrak{C}}({{\boldsymbol{\rho }}}_{\pm }(t))={[|{\rho }_{14}\pm {\rho }_{14}+{\rho }_{23}\mp {\rho }_{23}|-\sqrt{({\rho }_{11}^{-}\mp {\rho }_{11}^{-}+{\rho }_{22}^{+}\pm {\rho }_{22}^{+})({\rho }_{33}^{+}\pm {\rho }_{33}^{+}+{\rho }_{44}^{-}\mp {\rho }_{44}^{-})}]}^{+}.$$

For the initial input states (*t* = 0), the concurrences are given by

32 $${\mathfrak{C}}({{\boldsymbol{\rho }}}_{\pm }(0))=\frac{3\varepsilon -1}{2}\mathrm{.}$$

#### Quantum Discord

A computation of discord falls into the category of NP-complete problems in general^41^. However, the discord of the X-state can be determined analytically^35,42,43^. Letting ${\eta }_{i}^{\pm }(t)$, *i* = 1, 2, 3, 4, be the eigenvalues of the output Werner-like states ***ρ***_±_(*t*), the discord of ***ρ***_±_(*t*), denoted by ${\mathfrak{D}}({{\boldsymbol{\rho }}}_{\pm }(t))$, is given by

33 $${\mathfrak{D}}({{\boldsymbol{\rho }}}_{\pm }(t))=\,{\rm{\min }}\,\{{Q}_{1}^{\pm }(t),{Q}_{2}^{\pm }(t)\},$$

where

34 $${Q}_{1}^{\pm }(t)={\sum }_{i=1}^{4}{\eta }_{i}^{\pm }(t)\,{\mathrm{log}}_{2}{\eta }_{i}^{\pm }(t)+H({\rho }_{11}^{\pm }+{\rho }_{33}^{\pm })+H(\frac{1+\sqrt{{(1-2({\rho }_{33}^{\pm }+{\rho }_{44}^{\pm }))}^{2}+{|{\rho }_{14}\pm {\rho }_{14}+{\rho }_{23}\mp {\rho }_{23}|}^{2}}}{2}),$$

35 $${Q}_{2}^{\pm }(t)={\sum }_{i=1}^{4}({\eta }_{i}^{\pm }(t){\mathrm{log}}_{2}{\eta }_{i}^{\pm }(t)-{\rho }_{ii}^{\pm }\,{\mathrm{log}}_{2}{\rho }_{ii}^{\pm }),$$

with the Shannon entropy $H(p)=-\,p{\mathrm{log}}_{2}p-(1-p)\,{\mathrm{log}}_{2}(1-p)$. Note that the discord of a pure state coincides with the entanglement of formation and reaches its maximum value equal to one when the pure state is maximally entangled (*ε* = 1)^44,45^. For the initial input states (*t* = 0), we have

36 $${\mathfrak{D}}({{\boldsymbol{\rho }}}_{\pm }(0))=\frac{1}{4}(1-\varepsilon ){\mathrm{log}}_{2}(1-\varepsilon )+\frac{1}{4}(1+3\varepsilon ){\mathrm{log}}_{2}(1+3\varepsilon )-\frac{1}{2}(1+\varepsilon ){\mathrm{log}}_{2}(1+\varepsilon )\mathrm{.}$$

## Discussion

For the SGAD channel with correlated noise (*μ* = 1), the output states ***ρ***_±_(*t*) in (24) reduce to

37 $${{\boldsymbol{\rho }}}_{\pm }(t)=(\begin{array}{cccc}\frac{(2n+{\rm{\Lambda }}(t))(1\pm \varepsilon )}{4(2n+1)} & 0 & 0 & \frac{\varepsilon \sqrt{{\rm{\Lambda }}(t)}{e}^{-{\rm{\Omega }}mt}}{4}\pm \frac{\varepsilon \sqrt{{\rm{\Lambda }}(t)}{e}^{-{\rm{\Omega }}mt}}{4}\\ 0 & \frac{1\mp \varepsilon }{4} & \frac{\varepsilon \mp \varepsilon }{4} & 0\\ 0 & \frac{\varepsilon \mp \varepsilon }{4} & \frac{1\mp \varepsilon }{4} & 0\\ \frac{\varepsilon \sqrt{{\rm{\Lambda }}(t)}{e}^{-{\rm{\Omega }}mt}}{4}\pm \frac{\varepsilon \sqrt{{\rm{\Lambda }}(t)}{e}^{-{\rm{\Omega }}mt}}{4} & 0 & 0 & \frac{(2n+2-{\rm{\Lambda }}(t))(1\pm \varepsilon )}{4(2n+1)}\end{array})\mathrm{.}$$

In the steady state (*t* → ∞), the states ***ρ***_±_(*t*) have the form

38 $$\mathop{\mathrm{lim}}\limits_{t\to \infty }{{\boldsymbol{\rho }}}_{\pm }(t)=(\begin{array}{cccc}\tfrac{n[2n(1\pm \mu \varepsilon )+\mu (1\pm \varepsilon )]}{2{(2n+1)}^{2}} & 0 & 0 & 0\\ 0 & \tfrac{\mu (1\mp \varepsilon )+4n(1+n)(1\mp \mu \varepsilon )}{4{(2n+1)}^{2}} & \tfrac{\mu \varepsilon }{4}\mp \tfrac{\mu \varepsilon }{4} & 0\\ 0 & \tfrac{\mu \varepsilon }{4}\mp \tfrac{\mu \varepsilon }{4} & \tfrac{\mu (1\mp \varepsilon )+4n(1+n)(1\mp \mu \varepsilon )}{4{(2n+1)}^{2}} & 0\\ 0 & 0 & 0 & \tfrac{(n+1)[2(n+1)(1\pm \mu \varepsilon )-\mu (1\pm \varepsilon )]}{2{(2n+1)}^{2}}\end{array})\mathrm{.}$$

**Figure 1:**
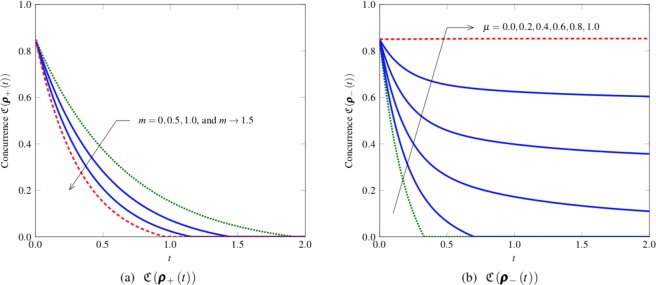
Concurrence dynamics (*n* = 1, Ω = 1, *ε* = 0.9): (**a**) ${\mathfrak{C}}({{\boldsymbol{\rho }}}_{+}(t))$ as a function of time *t* with *μ* = 1 when *m* = 0, 0.5, 1.0, and *m* → 1.5; and (**b**) ${\mathfrak{C}}({{\boldsymbol{\rho }}}_{-}(t))$ as a function of time *t* with *m* = 1 when *μ* = 0.0, 0.2, 0.4, 0.6, 0.8, and 1.0. The concurrence ${\mathfrak{C}}({{\boldsymbol{\rho }}}_{+}(t))$ decreases with *t*, and the ESD is appeared at ${t}_{\star }=1.927$, 1.445, 1.156, 0.963 for *m* = 0, 0.5, 1.0, and *m* → 1.5, respectively (see (41)). We can also observe that the decay of ${\mathfrak{C}}({{\boldsymbol{\rho }}}_{+}(t))$ over time becomes fast with increasing the squeezing parameter *m*. For the singlet-like state ***ρ***_−_(*t*), the entanglement can be preserved without the ESD due to the channel memory when *μ* > 0.344. In this example, ${\mathrm{lim}}_{t\to \infty }{\mathfrak{C}}({{\boldsymbol{\rho }}}_{-}(t))\mathrm{=0.073}$, 0.332, 0.592, and 0.853 for *μ* = 0.4, 0.6, 0.8, and 1.0, respectively (see (43)).

**Figure 2:**
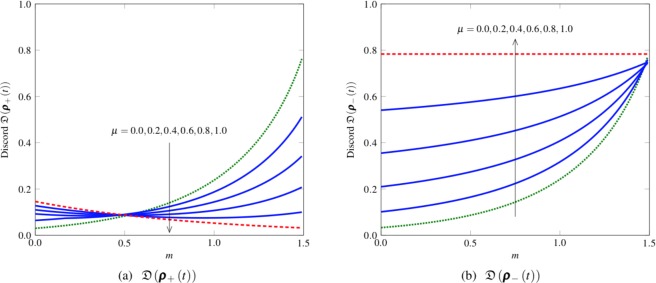
Discord dynamics (*n* = 1, Ω = 1, *ε* = 0.9): (**a**) ${\mathfrak{D}}({{\boldsymbol{\rho }}}_{+}(t))$ and (**b**) ${\mathfrak{D}}({{\boldsymbol{\rho }}}_{-}(t))$ at time *t* = 0.5 as a function of the squeezing parameter *m* when *μ* = 0.0, 0.2, 0.4, 0.6, 0.8, and 1.0. The squeezing effect reduces the decoherence^18^ and increases the discord, particularly under uncorrelated noise (*μ* = 0) or a low degree of channel memory. However, when *μ* = 1 (correlated noise), the discord remains constant for the singlet-like state ***ρ***_−_(*t*) or even slightly decreases for the state ***ρ***_+_(*t*) as the squeezing parameter *m* increases.

**Figure 3:**
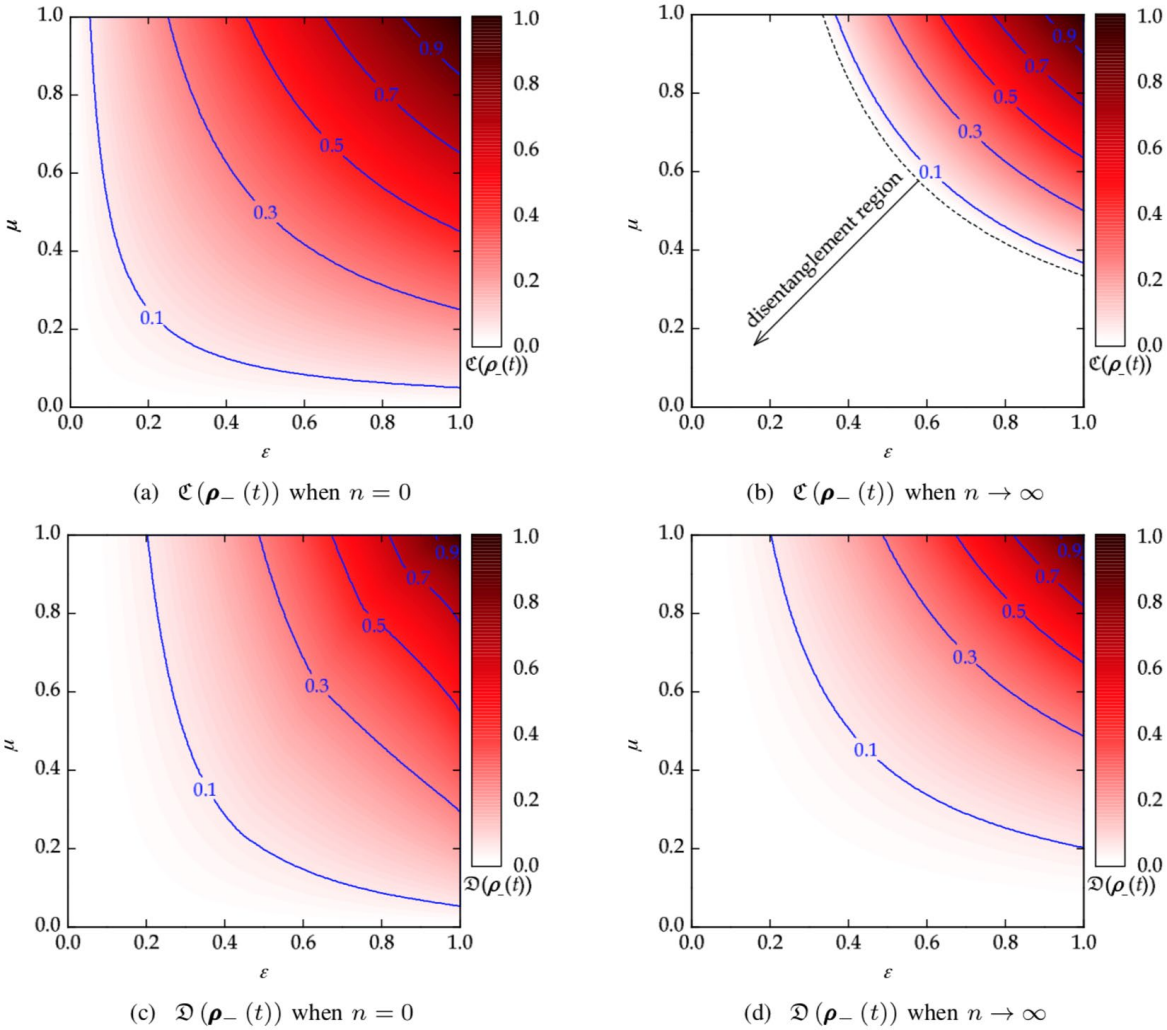
Quantum correlations in the steady state as a function of (*ε*, *μ*) for (**a**) ${\mathfrak{C}}({{\boldsymbol{\rho }}}_{-}(t))$ when *n* = 0, (**b**) ${\mathfrak{C}}({{\boldsymbol{\rho }}}_{-}(t))$ when *n* → ∞, (**c**) ${\mathfrak{D}}({{\boldsymbol{\rho }}}_{-}(t))$ when *n* = 0, and (**d**) ${\mathfrak{D}}({{\boldsymbol{\rho }}}_{-}(t))$ when *n* → ∞. The entanglement can be generated in the SGAD channel with memory even in case there is initially no entanglement between two qubits (*ε* < 1/3) when *n* = 0. However, as *n* → ∞, the state ***ρ***_−_(*t*) ends up with the disentanglement if *εμ* < 1/3 (see (44)). In other words, if the input state ***ρ***_−_(0) has no entanglement (*ε* < 1/3), then there is no entanglement generation even under correlated noise (*μ* = 1). The discord ${\mathfrak{D}}({{\boldsymbol{\rho }}}_{-}(t))$ in the steady state increases with *μ* and *ε*.

From (31–38), we make the following observations on the dynamics of concurrence and discord (see Figs 1–3).

- For correlated noise (*μ* = 1), the concurrences reduce to

39 $${\mathfrak{C}}({{\boldsymbol{\rho }}}_{+}(t))={[\varepsilon \sqrt{{\rm{\Lambda }}(t)}{e}^{-{\rm{\Omega }}mt}-\frac{1-\varepsilon }{2}]}^{+},$$

40 $${\mathfrak{C}}({{\boldsymbol{\rho }}}_{-}(t))={[\varepsilon -\frac{1-\varepsilon }{2(2n+1)}\sqrt{(2n+{\rm{\Lambda }}(t))(2n+2-{\rm{\Lambda }}(t))}]}^{+}\mathrm{.}$$

The concurrence ${\mathfrak{C}}({{\boldsymbol{\rho }}}_{+}(t))$ under correlated noise monotonically decreases with time *t* as well as the channel parameters (Ω, *n*, *m*) and ends up with the ESD at time

41 $${t}_{\star }=\frac{{\rm{l}}{\rm{n}}(\frac{2\varepsilon }{1-\varepsilon })}{{\rm{\Omega }}(n+m+1/2)}.$$

However, the squeezing parameter *m* does not affect to the dynamics of the concurrence and the discord for the singlet-like state ***ρ***_−_(*t*). For the maximally entangled state (*ε* = 1), the quantum correlations in the SGAD channel with correlated noise do not evolve in time and are preserved as ${\mathfrak{C}}({{\boldsymbol{\rho }}}_{-}(t))={\mathfrak{D}}({{\boldsymbol{\rho }}}_{-}(t))=1$.

- In the steady state (*t* → ∞), we have

42 $$\mathop{\mathrm{lim}}\limits_{t\to \infty }{\mathfrak{C}}({{\boldsymbol{\rho }}}_{+}(t))=\mathop{\mathrm{lim}}\limits_{t\to \infty }{\mathfrak{D}}({{\boldsymbol{\rho }}}_{+}(t))=\mathrm{0,}$$

43 $$\begin{array}{rcl}\mathop{\mathrm{lim}}\limits_{t\to \infty }{\mathfrak{C}}({{\boldsymbol{\rho }}}_{-}(t)) & = & [\varepsilon \mu -\frac{1}{2{(2n+1)}^{2}}\sqrt{4(1-\mu ){n}^{2}+2\mu n(2n+1)(1-\varepsilon )}\\  &  & {\times \sqrt{4(1-\mu ){(n+1)}^{2}+2\mu (n+1)(2n+1)(1-\varepsilon )}]}^{+}.\end{array}$$

The concurrence ${\mathfrak{C}}({{\boldsymbol{\rho }}}_{+}(t))$ and discord ${\mathfrak{D}}({{\boldsymbol{\rho }}}_{+}(t))$ vanishe with time regardless of the channel parameters and channel memory, while these quantum correlations for the singlet-like state ***ρ***_−_(*t*) can remain positive or be even generated depending on the channel parameters and memory as *t* → ∞. The quantum state ***ρ***_−_(*t*) looses the quantumness since the density matrix (38) in the steady state has only diagonal elements. For spontaneous emission (*n* = 0), the steady-state concurrence is frozen at ${\mathrm{lim}}_{t\to \infty }{\mathfrak{C}}({{\boldsymbol{\rho }}}_{-}(t))=\varepsilon \mu$. This means that the steady-state ${\mathfrak{C}}({{\boldsymbol{\rho }}}_{-}(t))$ increases with the degree of channel memory (*μ* > 0) and the correlated noise can even generate the entanglement for unentangled states (*ε* ≤ 1/3). As *n* → ∞, we have

44 $$\mathop{\mathrm{lim}}\limits_{t,n\to \infty }{\mathfrak{C}}({{\boldsymbol{\rho }}}_{-}(t))={[\frac{3\varepsilon \mu -1}{2}]}^{+},$$

which remains positive when *εμ* > 1/3.

## Methods

### Stochastic Map Φ_u_ for the SGAD Channel with Uncorrelated Noise

To find Φ_u_ for the SGAD channel with uncorrelated noise, we first consider the stochastic map Φ_1_ for a qubit of initial state $\rho \in {{\mathbb{C}}}^{2\times 2}$ sent by a single channel use, which is given at time *t* by introducing the Lindblad superoperator ${\mathcal L}$ as follows^17,21,29^:

45 $$\begin{array}{rcl}{{\rm{\Phi }}}_{1}(\rho ) & = & \exp ( {\mathcal L} t)\rho \\  & = & {\sum }_{i}{\rm{tr}}({L}_{i}\rho )\,\exp ({\lambda }_{i}t){R}_{i}\end{array}$$

46 $$\hspace{1.5cm}=\,{\sum }_{i}{\rm{t}}{\rm{r}}({R}_{i}\rho )\exp ({\lambda }_{i}t){L}_{i},$$

where *L*_*i*_ and *R*_*i*_ are the left and right eigenoperators of ${\mathcal L}$ in (3); *λ*_*i*_ are the corresponding eigenvalues; and tr(⋅) denotes the trace operator. The left and right eigenopertors satisfy the eigenvalue equations:

47 $$ {\mathcal L} {R}_{i}={\lambda }_{i}{R}_{i},$$

48 $${L}_{i} {\mathcal L} ={\lambda }_{i}{L}_{i},$$

which have the duality relation

49 $${\rm{tr}}({L}_{i}{R}_{i})={\delta }_{i,j}.$$

From (3) and (47–49), we can find the right eigenoperators *R*_*i*_ and the left eigenoperators *L*_*i*_ for the SGAD channel as^17^

50 $${R}_{1}=\frac{1}{\sqrt{2}}({\sigma }_{0}-\frac{1}{2n+1}{\sigma }_{3}),\,{L}_{1}=\frac{1}{\sqrt{2}}{\sigma }_{0},$$

51 $${R}_{2}={L}_{2}=\frac{1}{\sqrt{2}}({\sigma }_{+}+{\sigma }_{-}),\,{R}_{3}=-\,{L}_{3}=\frac{1}{\sqrt{2}}({\sigma }_{-}-{\sigma }_{+}),$$

52 $${R}_{4}=\frac{1}{\sqrt{2}}{\sigma }_{3},\,{L}_{4}=\frac{1}{\sqrt{2}}(\frac{1}{2n+1}{\sigma }_{0}+{\sigma }_{3}),$$

where *σ*_0_ = *I*, and the corresponding eigenvalues are given by

53 $$\{\begin{array}{l}{\lambda }_{1}=\mathrm{0,}\\ {\lambda }_{2}=-\,{\rm{\Omega }}(n+m+\mathrm{1/2}),\\ {\lambda }_{3}=-\,{\rm{\Omega }}(n-m+\mathrm{1/2}),\\ {\lambda }_{4}=-\,2{\rm{\Omega }}(n+\mathrm{1/2})\mathrm{.}\end{array}$$

Let

54 $$\rho =(\begin{array}{cc}{\rho }_{11} & {\rho }_{12}\\ {\rho }_{12}^{\ast } & {\rho }_{22}\end{array}),$$

where ${\rho }_{11},{\rho }_{22}\in {\mathbb{R}}$, *ρ*_11_ + *ρ*_22_ = 1, and ${\rho }_{12}\in {\mathbb{C}}$. Then, the output state Φ_1_(*ρ*) can be computed using (45) or (46) at time *t* as

55 $${{\rm{\Phi }}}_{1}(\rho )=(\begin{array}{cc}\tfrac{n}{2n+1}+\tfrac{1}{2}{e}^{-2{\rm{\Omega }}(n+\mathrm{1/2})t}({\rho }_{11}-{\rho }_{22}+\tfrac{1}{2n+1}) & {e}^{-{\rm{\Omega }}(n+\mathrm{1/2})t}({\rho }_{12}\,\cosh ({\rm{\Omega }}mt)-{\rho }_{12}^{\ast }\,\sinh ({\rm{\Omega }}mt))\\ {e}^{-{\rm{\Omega }}(n+\mathrm{1/2})t}({\rho }_{12}^{\ast }\,\cosh ({\rm{\Omega }}mt)-{\rho }_{12}\,\sinh ({\rm{\Omega }}mt)) & \tfrac{n+1}{2n+1}+\tfrac{1}{2}{e}^{-2{\rm{\Omega }}(n+\mathrm{1/2})t}(-{\rho }_{11}+{\rho }_{22}-\tfrac{1}{2n+1})\end{array})\mathrm{.}$$

Since the stochastic map (55) has a Kraus decomposition, we can obtain its Kraus operator-sum representation

56 $${{\rm{\Phi }}}_{1}(\rho )={\sum }_{i}{E}_{i}\rho {E}_{i}^{\dagger },$$

where the Kraus operators *E*_*i*_ are given in (6–11). Using these Kraus operators, we can obtain the stochastic map Φ_u_(***ρ***) for two qubits with initial states ${\boldsymbol{\rho }}\in {{\mathbb{C}}}^{4\times 4}$ sent over the memoryless SGAD channel as

57 $${{\rm{\Phi }}}_{{\rm{u}}}({\boldsymbol{\rho }})={\sum }_{j}(I\otimes {E}_{j})[{\sum }_{i}({E}_{i}\otimes I){\boldsymbol{\rho }}{({E}_{i}\otimes I)}^{\dagger }]{(I\otimes {E}_{j})}^{\dagger },$$

yielding the Kraus decomposition (5).

### Stochastic Map Φ_c_ for the SGAD Channel with Correlated Noise

To find Φ_c_ for the SGAD channel with correlated noise, we consider the following correlated version of the Lindblad:

58 $$\begin{array}{c}\tilde{ {\mathcal L} }{\boldsymbol{\rho }}=-\,\frac{{\rm{\Omega }}(n+1)}{2}({\sigma }_{+}^{\otimes 2}{\sigma }_{-}^{\otimes 2}{\boldsymbol{\rho }}+{\boldsymbol{\rho }}{\sigma }_{+}^{\otimes 2}{\sigma }_{-}^{\otimes 2}-2{\sigma }_{-}^{\otimes 2}{\boldsymbol{\rho }}{\sigma }_{+}^{\otimes 2})\\ \,\,-\frac{{\rm{\Omega }}n}{2}({\sigma }_{-}^{\otimes 2}{\sigma }_{+}^{\otimes 2}{\boldsymbol{\rho }}+{\boldsymbol{\rho }}{\sigma }_{-}^{\otimes 2}{\sigma }_{+}^{\otimes 2}-2{\sigma }_{+}^{\otimes 2}{\boldsymbol{\rho }}{\sigma }_{-}^{\otimes 2})-{\rm{\Omega }}m({\sigma }_{+}^{\otimes 2}{\boldsymbol{\rho }}{\sigma }_{+}^{\otimes 2}+{\sigma }_{-}^{\otimes 2}{\boldsymbol{\rho }}{\sigma }_{-}^{\otimes 2}),\end{array}$$

where ${\sigma }_{\pm }^{\otimes 2}={\sigma }_{\pm }\otimes {\sigma }_{\pm }$. The map Φ_c_(***ρ***) at time *t* is given by

59 $$\begin{array}{ccc}{{\rm{\Phi }}}_{{\rm{c}}}({\boldsymbol{\rho }}) & = & \exp (\mathop{{\mathscr{L}}}\limits^{ \sim }t){\boldsymbol{\rho }}\\  & = & {\sum }_{i}{\rm{t}}{\rm{r}}({\mathop{{\boldsymbol{L}}}\limits^{ \sim }}_{i}{\boldsymbol{\rho }})\,\exp ({\mathop{\lambda }\limits^{ \sim }}_{i}t){\mathop{{\boldsymbol{R}}}\limits^{ \sim }}_{i},\end{array}$$

where ${\tilde{{\boldsymbol{L}}}}_{{\boldsymbol{i}}}$ and ${\tilde{{\boldsymbol{R}}}}_{{\boldsymbol{i}}}$ are the left and right eigenoperators of $\tilde{ {\mathcal L} }$; and ${\tilde{\lambda }}_{i}$ are the corresponding eigenvalues. The right eigenopertors ${\tilde{{\boldsymbol{R}}}}_{{\boldsymbol{i}}}$ and the left eigenopertors ${\tilde{{\boldsymbol{L}}}}_{{\boldsymbol{i}}}$ are

60 $${\tilde{{\boldsymbol{R}}}}_{1}=\frac{1}{\sqrt{2}}(\begin{array}{cccc}\frac{2n}{2n+1} & 0 & 0 & 0\\ 0 & 0 & 0 & 0\\ 0 & 0 & 0 & 0\\ 0 & 0 & 0 & \frac{2(n+1)}{2n+1}\end{array}),\,{\tilde{{\boldsymbol{L}}}}_{1}=\frac{1}{\sqrt{2}}(\begin{array}{cccc}1 & 0 & 0 & 0\\ 0 & 0 & 0 & 0\\ 0 & 0 & 0 & 0\\ 0 & 0 & 0 & 1\end{array}),$$

61 $${\mathop{{\boldsymbol{R}}}\limits^{ \sim }}_{2}={\mathop{{\boldsymbol{L}}}\limits^{ \sim }}_{2}=(\begin{array}{cccc}0 & 0 & 0 & 0\\ 0 & 1 & 0 & 0\\ 0 & 0 & 0 & 0\\ 0 & 0 & 0 & 0\end{array}),\,{\mathop{{\boldsymbol{R}}}\limits^{ \sim }}_{3}={\mathop{{\boldsymbol{L}}}\limits^{ \sim }}_{3}=(\begin{array}{cccc}0 & 0 & 0 & 0\\ 0 & 0 & 0 & 0\\ 0 & 0 & 1 & 0\\ 0 & 0 & 0 & 0\end{array}),$$

62 $${\tilde{{\boldsymbol{R}}}}_{4}={\tilde{{\boldsymbol{L}}}}_{4}=\frac{1}{\sqrt{2}}(\begin{array}{cccc}0 & 0 & 0 & 0\\ 0 & 0 & 1 & 0\\ 0 & 1 & 0 & 0\\ 0 & 0 & 0 & 0\end{array}),\,{\tilde{{\boldsymbol{R}}}}_{5}=-\,{\tilde{{\boldsymbol{L}}}}_{5}=\frac{1}{\sqrt{2}}(\begin{array}{cccc}0 & 0 & 0 & 0\\ 0 & 0 & -\,1 & 0\\ 0 & 1 & 0 & 0\\ 0 & 0 & 0 & 0\end{array}),$$

63 $${\mathop{{\boldsymbol{R}}}\limits^{ \sim }}_{6}={\mathop{{\boldsymbol{L}}}\limits^{ \sim }}_{6}=\frac{1}{\sqrt{2}}(\begin{array}{cccc}0 & 1 & 0 & 0\\ 1 & 0 & 0 & 0\\ 0 & 0 & 0 & 0\\ 0 & 0 & 0 & 0\end{array}),\,{\mathop{{\boldsymbol{R}}}\limits^{ \sim }}_{7}=-\,{\mathop{{\boldsymbol{L}}}\limits^{ \sim }}_{7}=\frac{1}{\sqrt{2}}(\begin{array}{cccc}0 & -\,1 & 0 & 0\\ 1 & 0 & 0 & 0\\ 0 & 0 & 0 & 0\\ 0 & 0 & 0 & 0\end{array}),$$

64 $${\tilde{{\boldsymbol{R}}}}_{8}={\tilde{{\boldsymbol{L}}}}_{8}=\frac{1}{\sqrt{2}}(\begin{array}{cccc}0 & 0 & 1 & 0\\ 0 & 0 & 0 & 0\\ 1 & 0 & 0 & 0\\ 0 & 0 & 0 & 0\end{array}),\,{\tilde{{\boldsymbol{R}}}}_{9}=-\,{\tilde{{\boldsymbol{L}}}}_{9}=\frac{1}{\sqrt{2}}(\begin{array}{cccc}0 & 0 & -\,1 & 0\\ 0 & 0 & 0 & 0\\ 1 & 0 & 0 & 0\\ 0 & 0 & 0 & 0\end{array}),$$

65 $${\tilde{{\boldsymbol{R}}}}_{10}={\tilde{{\boldsymbol{L}}}}_{10}=\frac{1}{\sqrt{2}}(\begin{array}{cccc}0 & 0 & 0 & 1\\ 0 & 0 & 0 & 0\\ 0 & 0 & 0 & 0\\ 1 & 0 & 0 & 0\end{array}),\,{\tilde{{\boldsymbol{R}}}}_{11}=-\,{\tilde{{\boldsymbol{L}}}}_{11}=\frac{1}{\sqrt{2}}(\begin{array}{cccc}0 & 0 & 0 & -\,1\\ 0 & 0 & 0 & 0\\ 0 & 0 & 0 & 0\\ 1 & 0 & 0 & 0\end{array}),$$

66 $${\tilde{{\boldsymbol{R}}}}_{12}={\tilde{{\boldsymbol{L}}}}_{12}=\frac{1}{\sqrt{2}}(\begin{array}{cccc}0 & 0 & 0 & 0\\ 0 & 0 & 0 & 1\\ 0 & 0 & 0 & 0\\ 0 & 1 & 0 & 0\end{array}),\,{\tilde{{\boldsymbol{R}}}}_{13}=-\,{\tilde{{\boldsymbol{L}}}}_{13}=\frac{1}{\sqrt{2}}(\begin{array}{cccc}0 & 0 & 0 & 0\\ 0 & 0 & 0 & -\,1\\ 0 & 0 & 0 & 0\\ 0 & 1 & 0 & 0\end{array}),$$

67 $${\tilde{{\boldsymbol{R}}}}_{14}={\tilde{{\boldsymbol{L}}}}_{14}=\frac{1}{\sqrt{2}}(\begin{array}{cccc}0 & 0 & 0 & 0\\ 0 & 0 & 0 & 0\\ 0 & 0 & 0 & 1\\ 0 & 0 & 1 & 0\end{array}),\,{\tilde{{\boldsymbol{R}}}}_{15}=-\,{\tilde{{\boldsymbol{L}}}}_{15}=\frac{1}{\sqrt{2}}(\begin{array}{cccc}0 & 0 & 0 & 0\\ 0 & 0 & 0 & 0\\ 0 & 0 & 0 & -\,1\\ 0 & 0 & 1 & 0\end{array}),$$

68 $${\tilde{{\boldsymbol{R}}}}_{16}=\frac{1}{\sqrt{2}}(\begin{array}{cccc}1 & 0 & 0 & 0\\ 0 & 0 & 0 & 0\\ 0 & 0 & 0 & 0\\ 0 & 0 & 0 & -\,1\end{array}),\,{\tilde{{\boldsymbol{L}}}}_{16}=\frac{1}{\sqrt{2}}(\begin{array}{cccc}\frac{2(n+1)}{2n+1} & 0 & 0 & 0\\ 0 & 0 & 0 & 0\\ 0 & 0 & 0 & 0\\ 0 & 0 & 0 & -\frac{2n}{2n+1}\end{array}),$$

and the corresponding eigenvalues are given by

69 $$(\begin{array}{l}{\tilde{\lambda }}_{1}={\tilde{\lambda }}_{2}={\tilde{\lambda }}_{3}={\tilde{\lambda }}_{4}={\tilde{\lambda }}_{5}=0,\\ {\tilde{\lambda }}_{6}={\tilde{\lambda }}_{7}={\tilde{\lambda }}_{8}={\tilde{\lambda }}_{9}=-\,{\rm{\Omega }}(n+1)/2,\\ {\tilde{\lambda }}_{10}=-\,{\rm{\Omega }}(n+m+1/2),\\ {\tilde{\lambda }}_{11}=-\,{\rm{\Omega }}(n-m+1/2),\\ {\tilde{\lambda }}_{12}={\tilde{\lambda }}_{13}={\tilde{\lambda }}_{14}={\tilde{\lambda }}_{15}=-\,{\rm{\Omega }}n/2,\\ {\tilde{\lambda }}_{16}=-\,2{\rm{\Omega }}(n+1/2).\end{array}$$

The stochastic map (59) has the Kraus decomposition (12).

## Supplementary information

Supplementary Material
