# Supplementary material for: Quantum Correlation in Squeezed Generalized Amplitude Damping Channels with Memory
Source: Sci Rep. 2019 Mar 11;9:4035. doi: 10.1038/s41598-019-40652-0 (PMC6411861; doi:10.1038/s41598-019-40652-0)
Supplement: Supplementary file 1 — Supplementary Material [file 41598_2019_40652_MOESM1_ESM.pdf]

Supplementary Material: Quantum Correlation in Squeezed Generalized Amplitude Damping Channels with Memory

Youngmin Jeong and Hyundong Shin

Department of Electronic Engineering, Kyung Hee University, Yongin-si, 17104 Korea
Correspondence and request for materials should be addressed to H.S (email: hshin@khu.ac.kr)

Example 1 (Unital and Nonunital Quantum Channels)

To highlight a Kraus decomposition of the stochastic map for a quantum channel with correlated noise into a tensorial or non-tensorial form, we provide two simple examples for unital and nonunital quantum channels[1–6]. We first consider the dephasing (phase damping) channel with memory as a unital example. In this case, the Kraus operators E_i for Φ_1 are the Pauli operators, i.e., $E_1 = \sqrt{p_0}\sigma_0$ and $E_2 = \sqrt{p_3}\sigma_3$, where $p_0 = 1-p$, $p_3 = p$, and $p = \frac{1}{2}\left(1-e^{-\Gamma t}\right)$ with the damping parameter Γ. To find $\Phi_c(\boldsymbol{\rho})$ for the dephasing channel with correlated noise, we consider the following Lindblad:

$$\tilde{\mathscr{L}}\boldsymbol{\rho} = -\frac{\Gamma}{2}\left(\boldsymbol{\rho} - \sigma_3^{\otimes 2}\boldsymbol{\rho}\sigma_3^{\otimes 2}\right). \tag{S1}$$

With the eigenoperators $\tilde{\boldsymbol{R}}_{ij} = \tilde{\boldsymbol{L}}_{ij} = \frac{1}{2}\sigma_i \otimes \sigma_j$, $i = 0,1,2,3$, and the corresponding eigenvalues

$$\begin{cases} \tilde{\lambda}_{00} = \tilde{\lambda}_{11} = \tilde{\lambda}_{22} = \tilde{\lambda}_{33} = \tilde{\lambda}_{03} = \tilde{\lambda}_{30} = \tilde{\lambda}_{12} = \tilde{\lambda}_{21} = 0, \\ \tilde{\lambda}_{01} = \tilde{\lambda}_{10} = \tilde{\lambda}_{02} = \tilde{\lambda}_{20} = \tilde{\lambda}_{13} = \tilde{\lambda}_{31} = \tilde{\lambda}_{23} = \tilde{\lambda}_{32} = -\Gamma, \end{cases} \tag{S2}$$

we can obtain

$$\begin{aligned} \Phi_c(\boldsymbol{\rho}) &= \sum_{i,j=0}^{3} \operatorname{tr}\left(\tilde{\boldsymbol{L}}_{ij}\boldsymbol{\rho}\right)\exp\left(-\tilde{\lambda}_{ij}t\right)\tilde{\boldsymbol{R}}_{ij} \\ &= \sum_{k\in\{0,3\}} p_k\left(\sigma_k\otimes\sigma_k\right)\boldsymbol{\rho}\left(\sigma_k\otimes\sigma_k\right)^{\dagger}. \end{aligned} \tag{S3}$$

Hence, the complete action $\Phi(\boldsymbol{\rho})$ for the dephasing channel with memory can be written as

$$\Phi(\boldsymbol{\rho}) = (1-\mu)\sum_{i,j=1}^{2}\boldsymbol{A}_{i,j}\boldsymbol{\rho}\boldsymbol{A}_{i,j}^{\dagger} + \mu\sum_{k\in\{0,3\}}\boldsymbol{B}_k\boldsymbol{\rho}\boldsymbol{B}_k^{\dagger}, \tag{S4}$$

where $\boldsymbol{A}_{i,j} = E_i \otimes E_j$ and $\boldsymbol{B}_k = \sqrt{p_k}\left(\sigma_k\otimes\sigma_k\right)$ are of tensorial form.

We next consider the amplitude damping channel with memory as a nonunital example. Since this channel is equivalent to the SGAD channel by setting $n = m = 0$, the Kraus operators E_i for Φ_1 in (56) of the main text and $\boldsymbol{B}_k$ for Φ_c in (12) of the main text reduce to

$$E_1 = \begin{pmatrix} \sqrt{1-q-\sqrt{1-q}} & 0 \\ 0 & 1 \end{pmatrix}, \tag{S5}$$

$$E_2 = \begin{pmatrix} 0 & 0 \\ \sqrt{q} & 0 \end{pmatrix}, \tag{S6}$$

$$E_3 = \begin{pmatrix} 0 & 0 \\ 0 & \sqrt{1-\sqrt{1-q}} \end{pmatrix}, \tag{S7}$$

$$E_4 = \begin{pmatrix} \sqrt[4]{1-q} & 0 \\ 0 & \sqrt[4]{1-q} \end{pmatrix}, \tag{S8}$$

$$\boldsymbol{B}_1 = \begin{pmatrix} \sqrt{1-q} & 0 & 0 & 0 \\ 0 & 1 & 0 & 0 \\ 0 & 0 & 1 & 0 \\ 0 & 0 & 0 & 1 \end{pmatrix}, \tag{S9}$$

$$\boldsymbol{B}_2 = \begin{pmatrix} 0 & 0 & 0 & 0 \\ 0 & 0 & 0 & 0 \\ 0 & 0 & 0 & 0 \\ \sqrt{q} & 0 & 0 & 0 \end{pmatrix}, \tag{S10}$$

where $q = 1 - e^{-\Omega t}$ with the damping parameter Ω, which is the Einstein coefficient of spontaneous emission. Hence, the stochastic map $\Phi(\boldsymbol{\rho})$ for the amplitude damping channel with memory is given by

$$\Phi(\boldsymbol{\rho}) = (1-\mu) \sum_{i,j=1}^{4} \boldsymbol{A}_{i,j} \boldsymbol{\rho} \boldsymbol{A}_{i,j}^{\dagger} + \mu \sum_{k=1}^{2} \boldsymbol{B}_k \boldsymbol{\rho} \boldsymbol{B}_k^{\dagger}, \tag{S11}$$

where $\boldsymbol{A}_{i,j} = E_i \otimes E_j$. Note that the Kraus operator $\boldsymbol{B}_2$ is not of tensorial form. This can be interpreted as a *spooky action* of the amplitude damping channel with memory[1]. The amplitude damping channel with correlated noise only allows the synchronous transition of the two qubits in contrast to the amplitude damping channel with uncorrelated noise, where the two qubits decay independently.

Example 2 (X-States)

Consider a density matrix $\boldsymbol{\rho}_{\mathrm{X}}$ of the form[7]

$$\boldsymbol{\rho}_{\mathrm{X}} = \begin{pmatrix} \rho_{11} & 0 & 0 & \rho_{14} \\ 0 & \rho_{22} & \rho_{23} & 0 \\ 0 & \rho_{23}^* & \rho_{33} & 0 \\ \rho_{14}^* & 0 & 0 & \rho_{44} \end{pmatrix}, \tag{S12}$$

which is called X-states in the computational basis $|00\rangle$, $|01\rangle$, $|10\rangle$, and $|11\rangle$ for two qubits where $\mathrm{tr}(\boldsymbol{\rho}_{\mathrm{X}}) = 1$, and $\rho_{14}, \rho_{23} \in \mathbb{C}$. Then, the stochastic map $\Phi_{\mathrm{c}}(\boldsymbol{\rho}_{\mathrm{X}})$ for the correlated action is given by

$$\Phi_{\mathrm{c}}(\boldsymbol{\rho}_{\mathrm{X}}) = \begin{pmatrix} \rho_{\mathrm{c}11} & 0 & 0 & \rho_{\mathrm{c}14} \\ 0 & \rho_{\mathrm{c}22} & \rho_{\mathrm{c}23} & 0 \\ 0 & \rho_{\mathrm{c}23}^* & \rho_{\mathrm{c}33} & 0 \\ \rho_{\mathrm{c}14}^* & 0 & 0 & \rho_{\mathrm{c}44} \end{pmatrix}, \tag{S13}$$

with the elements

$$\rho_{\mathrm{c}11} = \frac{n(\rho_{11}+\rho_{44}) + ((n+1)\rho_{11} - n\rho_{44})\Lambda(t)}{2n+1}, \tag{S14}$$

$$\rho_{\mathrm{c}22} = \rho_{22}, \tag{S15}$$

$$\rho_{\mathrm{c}33} = \rho_{33}, \tag{S16}$$

$$\rho_{\mathrm{c}44} = \frac{(n+1)(\rho_{11}+\rho_{44}) - ((n+1)\rho_{11} - n\rho_{44})\Lambda(t)}{2n+1}, \tag{S17}$$

$$\rho_{\mathrm{c}14} = \sqrt{\Lambda(t)} e^{-\Omega m t} \Re(\rho_{14}) + \imath \sqrt{\Lambda(t)} e^{\Omega m t} \Im(\rho_{14}), \tag{S18}$$

$$\rho_{\mathrm{c}23} = \rho_{23}, \tag{S19}$$

where $\Lambda(t) = e^{-\Omega(2n+1)t}$ is the damping parameter and $\imath = \sqrt{-1}$. The stochastic map $\Phi_{\mathrm{u}}(\boldsymbol{\rho}_{\mathrm{X}})$ for the uncorrelated action is

$$\Phi_{\mathrm{u}}(\boldsymbol{\rho}_{\mathrm{X}}) = \begin{pmatrix} \rho_{\mathrm{u}11} & 0 & 0 & \rho_{\mathrm{u}14} \\ 0 & \rho_{\mathrm{u}22} & \rho_{\mathrm{u}23} & 0 \\ 0 & \rho_{\mathrm{u}23}^* & \rho_{\mathrm{u}33} & 0 \\ \rho_{\mathrm{u}14}^* & 0 & 0 & \rho_{\mathrm{u}44} \end{pmatrix}, \tag{S20}$$

where the elements are given by

$$\rho_{u11} = \frac{n^2 + n\Lambda(t) - n(n+1)\Lambda^2(t)}{(2n+1)^2} + \frac{n\Lambda(t) + (n+1)\Lambda^2(t)}{2n+1}\rho_{11} - \frac{n\Lambda(t) - n\Lambda^2(t)}{2n+1}\rho_{44}, \tag{S21}$$

$$\begin{aligned}\rho_{u22} = {} & \frac{n(n+1)}{(2n+1)^2} + \frac{(n+1)\Lambda(t) - (n+1)^2\Lambda^2(t)}{(2n+1)^2}\rho_{11} + \frac{\left(2n^2+2n+1\right)\Lambda(t) + n(n+1)\Lambda^2(t)}{(2n+1)^2}\rho_{22} \\ & - \frac{2n(n+1)\Lambda(t) - n(n+1)\Lambda^2(t)}{(2n+1)^2}\rho_{33} - \frac{n\Lambda(t) + n^2\Lambda^2(t)}{(2n+1)^2}\rho_{44},\end{aligned} \tag{S22}$$

$$\begin{aligned}\rho_{u33} = {} & \frac{n(n+1)}{(2n+1)^2} + \frac{(n+1)\Lambda(t) - (n+1)^2\Lambda^2(t)}{(2n+1)^2}\rho_{11} - \frac{2n(n+1)\Lambda(t) - n(n+1)\Lambda^2(t)}{(2n+1)^2}\rho_{22} \\ & + \frac{\left(2n^2+2n+1\right)\Lambda(t) + n(n+1)\Lambda^2(t)}{(2n+1)^2}\rho_{33} - \frac{n\Lambda(t) + n^2\Lambda^2(t)}{(2n+1)^2}\rho_{44},\end{aligned} \tag{S23}$$

$$\rho_{u44} = \frac{(n+1)^2 - (n+1)\Lambda(t) - n(n+1)\Lambda^2(t)}{(2n+1)^2} - \frac{(n+1)\Lambda(t) - (n+1)\Lambda^2(t)}{2n+1}\rho_{11} + \frac{(n+1)\Lambda(t) + n\Lambda^2(t)}{2n+1}\rho_{44}, \tag{S24}$$

$$\rho_{u14} = \Lambda(t)\Big(-\imath\Im(\rho_{14}) + \Re(\rho_{14})\cosh(2\Omega mt) - \Re(\rho_{23})\sinh(2\Omega mt)\Big), \tag{S25}$$

$$\rho_{u23} = \Lambda(t)\Big(-\imath\Im(\rho_{23}) + \Re(\rho_{23})\cosh(2\Omega mt) - \Re(\rho_{14})\sinh(2\Omega mt)\Big). \tag{S26}$$

References

1. Yeo, Y. & Skeen, A. Time-correlated quantum amplitude-damping channel. *Phys. Rev. A* **67**, 064301 (2003).

2. Daffer, S., Wódkiewicz, K. & McIver, J. K. Quantum Markov channels for qubits. *Phys. Rev. A* **67**, 062312 (2003).

3. Macchiavello, C. & Palma, G. M. Entanglement-enhanced information transmission over a quantum channel with correlated noise. *Phys. Rev. A* **65**, 050301 (2002).

4. Macchiavello, C. & Sacchi, M. F. Witnessing quantum capacities of correlated channels. *Phys. Rev. A* **94**, 052333 (2016).

5. Xiao, X., Yao, Y., Xie, Y.-M., Wang, X.-H. & Li, Y.-L. Protecting entanglement from correlated amplitude damping channel using weak measurement and quantum measurement reversal. *Quantum Inf. Process.* **15**, 3881–3891 (2016).

6. Guo, Y.-N., Fang, M.-F., Wang, G.-Y. & Zeng, K. Generation and protection of steady-state quantum correlations due to quantum channels with memory. *Quantum Inf. Process.* **16**, 5129–5144 (2016).

7. Yu, T. & Eberly, J. H. Evolution from entanglement to decoherence of bipartite mixed "X" states. *Quantum Inf. Comput.* **7**, 459–468 (2007).
